# Exposure–response analyses for the MET inhibitor tepotinib including patients in the pivotal VISION trial: support for dosage recommendations

**DOI:** 10.1007/s00280-022-04441-3

**Published:** 2022-06-30

**Authors:** Wenyuan Xiong, Sofia Friberg Hietala, Joakim Nyberg, Orestis Papasouliotis, Andreas Johne, Karin Berghoff, Kosalaram Goteti, Jennifer Dong, Pascal Girard, Karthik Venkatakrishnan, Rainer Strotmann

**Affiliations:** 1 Merck Institute of Pharmacometrics, Lausanne, Switzerland; 2 Pharmetheus AB, Uppsala, Sweden; 3 Merck Healthcare KGaA, Darmstadt, Germany; 4 EMD Serono Research and Development Institute Inc., Billerica, MA USA

**Keywords:** Tyrosine kinase inhibitor, Dose selection, Targeted therapies, NSCLC, *MET*ex14 skipping alteration

## Abstract

**Purpose:**

Tepotinib is a highly selective MET inhibitor approved for treatment of non-small cell lung cancer (NSCLC) harboring *MET*ex14 skipping alterations. Analyses presented herein evaluated the relationship between tepotinib exposure, and efficacy and safety outcomes.

**Methods:**

Exposure–efficacy analyses included data from an ongoing phase 2 study (VISION) investigating 500 mg/day tepotinib in NSCLC harboring *MET*ex14 skipping alterations. Efficacy endpoints included objective response, duration of response, and progression-free survival. Exposure–safety analyses included data from VISION, plus four completed studies in advanced solid tumors/hepatocellular carcinoma (30–1400 mg). Safety endpoints included edema, serum albumin, creatinine, amylase, lipase, alanine aminotransferase, aspartate aminotransferase, and QT interval corrected using Fridericia’s method (QTcF).

**Results:**

Tepotinib exhibited flat exposure–efficacy relationships for all endpoints within the exposure range observed with 500 mg/day. Tepotinib also exhibited flat exposure–safety relationships for all endpoints within the exposure range observed with 30–1400 mg doses. Edema is the most frequently reported adverse event and the most frequent cause of tepotinib dose reductions and interruptions; however, the effect plateaued at low exposures. Concentration-QTc analyses using data from 30 to 1400 mg tepotinib resulted in the upper bounds of the 90% confidence interval being less than 10 ms for the mean exposures at the therapeutic (500 mg) and supratherapeutic (1000 mg) doses.

**Conclusions:**

These analyses provide important quantitative pharmacologic support for benefit/risk assessment of the 500 mg/day dosage of tepotinib as being appropriate for the treatment of NSCLC harboring *MET*ex14 skipping alterations.

**Registration Numbers:**

NCT01014936, NCT01832506, NCT01988493, NCT02115373, NCT02864992.

**Supplementary Information:**

The online version contains supplementary material available at 10.1007/s00280-022-04441-3.

## Introduction

Tepotinib is an oral, highly selective MET inhibitor approved in Brazil, Canada, Great Britain, Japan, Switzerland, Taiwan, and the USA for the treatment of patients with unresectable, advanced or metastatic non-small cell lung cancer (NSCLC) and *MET* exon 14 (*MET*ex14) skipping alterations. Recent guidelines for the treatment of NSCLC recommend tepotinib as a preferred first-line monotherapy option for patients with metastatic NSCLC and *MET*ex14 skipping alterations [1, 2].

MET is a tyrosine kinase receptor expressed by epithelial cells, neurons, hepatocytes, and hematopoietic cells [3, 4]. Activation by the ligand, hepatocyte growth factor (HGF), induces MET receptor dimerization and phosphorylation of tyrosine residues in the cytoplasmic tail of the receptor that engages with intracellular signaling pathways [3, 4]. Mutations in the *MET* splicing regions for exon 14 can lead to exon 14 skipping and the resulting translation of a shortened MET receptor, which lacks the juxtamembrane domain of the cytoplasmic tail [3–5]. The resulting aberrant HGF-MET signaling is involved in oncogenesis, promoting tumor proliferation, invasive growth, and angiogenesis.

Clinical evaluation of tepotinib 500 mg/day in patients with advanced NSCLC and confirmed *MET*ex14 skipping alterations is continuing with the ongoing phase 2 VISION study (NCT02864992) [6]. In the primary analysis of the VISION study, which assessed efficacy in patients with ≥ 9 months’ follow-up as of January 1, 2020, the objective response (OR) rate (by independent review) was 46% (95% confidence interval [CI] 36, 57) and the median duration of response (DOR, based on Kaplan–Meier [KM] analysis) was 11.1 months (95% CI 7.2, not estimable) [6]. In this study, 28% of all patients receiving tepotinib had grade ≥ 3 treatment-related adverse events (AEs) and 11% of patients had treatment-related AEs that led to permanent discontinuation of treatment. Treatment-related peripheral edema was the most common grade ≥ 3 toxicity, occurring in 7% of patients, and leading to treatment discontinuation in 5% of patients.

Tepotinib has shown activity in preclinical models of cancer [7–10] and promising anti-cancer activity in patients with MET-driven tumors [11–13]. In NSCLC with *MET* amplification-driven resistance to epidermal growth factor receptor (EGFR) inhibitors, the combination of tepotinib plus gefitinib showed anti-tumor activity in the INSIGHT study [13], and clinical activity of tepotinib plus osimertinib is being evaluated in the INSIGHT 2 study (NCT03940703).

The maximum tolerated dose of tepotinib was not reached in the first-in-human dose ranging study, which evaluated the safety profile of tepotinib at doses between 30 and 1400 mg/day [11]. A tepotinib dose of 500 mg/day (as hydrochloride hydrate, equivalent to 450 mg/day tepotinib free base) was subsequently established as the recommended phase 2 dose, using a translational modeling approach that integrated clinical and non-clinical pharmacokinetic (PK) and tumor pharmacodynamic (PD) data, and non-clinical efficacy data [14]. This model suggested that a once daily tepotinib dose of 500 mg will achieve plasma concentrations at or above the PD threshold of close-to-complete tumor phospho-MET inhibition (≥ 95%) in at least 90% of patients.

The aims of the present analyses were to evaluate the relationship between the exposure of tepotinib, and efficacy and safety outcomes following tepotinib administration. The relationship between the exposure of the major circulating human metabolite MSC2571109A, and safety outcomes was also evaluated. MSC2571109A is not thought to contribute to the efficacy of tepotinib based on preclinical PK/efficacy, and clinical PK profiling. The influence of potential covariates on exposure–efficacy analyses (OR, DOR, and progression-free survival [PFS]) was evaluated in patients with NSCLC harboring *MET*ex14 skipping alterations from the VISION study [6]. In summary, the analysis is considered comprehensive to offer a holistic view on the benefit/risk profile across the attained exposure, confirming the relevance of the clinical dose in the target population.

## Materials and methods

### Exposure–efficacy analyses

#### Study design and patient population

VISION (NCT02864992) is an ongoing, multicenter, phase 2, single-arm study of patients with histologically or cytologically confirmed advanced (stage IIIB/IV) NSCLC with measurable disease (confirmed by independent review committee per Response Evaluation Criteria in Solid Tumors [RECIST] 1.1) and *MET*ex14 skipping alterations (cohorts A and C) or *MET* amplification (cohort B), based on liquid or tumor biopsy [6]. All patients are receiving oral tepotinib 500 mg once daily until disease progression, death, or undue toxicity. The primary endpoint is OR assessed by an independent review committee; secondary endpoints include investigator-assessed OR, DOR, and PFS. Overall survival is also a secondary endpoint, but this endpoint was not included in the current analyses. The present exposure–efficacy analyses were based on all patients from cohort A who had completed a minimum of 9 months' follow-up from start of treatment at the time of data cut-off (July 1, 2020) [15].

#### Analyses

The relationship between tepotinib exposure and the efficacy outcomes, OR, DOR, and PFS was evaluated using tepotinib 24-h area under the concentration–time curve at steady state (AUC_τ,ss_) as the exposure metric. Individual tepotinib AUC_τ,ss_ was predicted from a tepotinib population PK model [14]. Other exposure metrics, AUC_0-24_ on day one of treatment and mean daily AUC until the first confirmed best overall response, were explored in an earlier analysis version based on a subset of the patients, but did not result in meaningful differences in the exposure–response association (data on file).

Efficacy endpoints were stratified by tepotinib exposure quartile. The relationship between OR and AUC_τ,ss_ was examined graphically by estimating OR and the corresponding 2-sided exact Clopper–Pearson 95% CIs for each quartile of tepotinib AUC_τ,ss_. Relationships between tepotinib exposure and DOR or PFS were visualized using KM curves stratified by exposure quartile. The influence of covariates and tepotinib exposure AUC_τ,ss_ on the OR was assessed using the full fixed effects model approach [16].

### Exposure–safety analyses

#### Study design and patient population

The data for safety analyses were based on all patients who received 30–1400 mg/day tepotinib monotherapy in four completed studies and the ongoing VISION study (Table 1). These studies included two phase 1 dose-finding studies in patients with advanced solid tumors (referred to herein as studies 001 and 003) [11, 12]; two phase 1b/2 studies that were conducted in patients with advanced hepatocellular carcinoma (HCC) (studies 004 and 005) [17, 18], and the ongoing phase 2 VISION study in patients with advanced NSCLC and *MET*ex14 skipping alterations or *MET* amplification (study 022) [6]. All completed studies used the data cut-off for the final analyses and the VISION study used the January 1, 2020 data cut-off. Data from serial samples were also assessed to evaluate the time course of change in serum creatinine in healthy volunteers following a single dose of tepotinib 500 mg (study 007) [19].

**Table 1:** Study design summary

Study number	001 (NCT01014936) [11]	003 (NCT01832506) [12]	004 (NCT01988493) [17]	005 (NCT02115373) [18]	007 (EudraCT 2013–003226-86) [19]	VISION; 022 (NCT02864992) [6]
Title	A phase 1 open label, non-randomized, dose-escalation first-in-man trial to investigate the c-Met kinase inhibitor MSC2156119J under three different regimens in subjects with advanced solid tumors	A Japanese multicenter, open label, phase 1 trial of c-Met inhibitor MSC2156119J given orally as monotherapy to subjects with solid tumors	A multicenter, randomized, phase 1b/2 trial to evaluate the efficacy, safety, and PK of MSC2156119J as monotherapy versus sorafenib in Asian subjects with MET + advanced hepatocellular carcinoma and Child–Pugh class A liver function	A multicenter, single-arm, phase 1b/2 study to evaluate efficacy, safety, and PK of MSC2156119J as monotherapy in subjects with MET + advanced hepatocellular carcinoma with Child Pugh class A liver function who have failed sorafenib treatment	A phase 1, pen label, three-part, single-center trial to investigate the absolute and relative bioavailability, mass balance, and metabolite profile of MSC2156119J in healthy male subjects	A phase 2 single-arm trial to investigate tepotinib in advanced (locally advanced or metastatic) non-small cell lung cancer with *MET* exon 14 (*MET*ex14) skipping alterations or *MET* amplification (VISION)
Methodology and study design	Phase 1, multicenter, open label, non-randomized, sequential and parallel group study	Phase 1, multicenter, open label, non-randomized, sequential group study	Phase 1b/2 multicenter, open label, single arm (phase 1b) and randomized, active-controlled (phase 2) study	Phase 1b/2 multicenter, open label, single-arm study	Phase 1, single-center, open label, parallel group study	Phase 2 multicenter, open label, single-arm study
Participants	149 adult patients with advanced solid tumors	12 Japanese adult patients with solid tumors	72 Asian, adult patients with advanced HCC and Child–Pugh class A liver function treated with tepotinib	66 adult patients with advanced HCC and Child–Pugh class A liver function	27 healthy participants	206 adult patients with advanced (stage IIIB/IV) NSCLC with *MET* exon 14 skipping alterations or *MET* amplification
Tepotinib treatment	Tepotinib doses of 30 mg to 1400 mg/day in three different treatment regimens (QD, 2 weeks on - 1 week off, TIW)	Tepotinib 215, 300, or 500 mg/day QD	Tepotinib 300, 500, 1000 mg/day QD	Tepotinib 300 or 500 mg/day QD	Tepotinib 100 mg or 500 mg single dose	Tepotinib 500 mg/day QD over 21-day cycle(s) until disease progression or undue toxicity
Number of patients and events included in present analyses						
*Efficacy*						
Overall	0	0	0	0	0	152
OR	0	0	0	0	0	146
DOR	0	0	0	0	0	66
PFS	0	0	0	0	0	146
*Safety*						
Number of participants in analysis set	149	12	72	66	27	206
Edema event						
Patients	33	4	32	40	0	130
Observations	48	4	60	71	0	313
Serum albumin evaluation						
Patients	149	12	72	64	0	201
Observations	1716	153	804	648	0	2015
Serum creatinine evaluation						
Patients	149	12	72	65	11^a^	201
Observations	1739	153	825	745	56	2068
QTcF interval						
Patients	144	12	70	59	0	107
Observations	953	91	568	504	0	1083

*DOR*, duration of response; *HCC*, hepatocellular carcinoma; *NSCLC*, non-small cell lung cancer; *OR*, objective response; *PFS*, progression-free survival; *PK*, pharmacokinetics; *QD*, once daily; *QTcF*, QT interval corrected using Fredericia’s formula; *TIW*, three times a week

^a^Serial-sampled serum creatinine data from study 007 were used to illustrate the time-profile of serum creatinine

#### Analyses

Safety endpoints of identified risks assessed were: edema (time-to-first event and maximum severity grade, based on a composite endpoint that included the terms: face edema, edema, edema peripheral, localized edema, edema genital, periorbital edema, scrotal edema, peripheral swelling and abdominal wall edema), serum albumin, creatinine, amylase, lipase, alanine aminotransferase (ALT), aspartate aminotransferase (AST), and QTc interval. The relationship between tepotinib exposure and edema was evaluated using different exposure metrics, mean tepotinib AUC_0-24_ in the week before the edema event or mean AUC_0-24_ until the event in the visual exploratory analyses, and time-varying daily AUC_24h_ for the edema time-to-event (TTE) and longitudinal albumin modeling. The relationships between exposure of tepotinib or MSC2571109A, and grade ≥ 3 AE (as defined by Common Terminology Criteria for Adverse Events [CTCAE] v4.03 [20]), treatment discontinuation due to an AE, and dose reduction due to an AE were also evaluated.

An exploratory graphical analysis was performed for each safety endpoint of interest to evaluate the potential association to tepotinib exposure quartile. The relationships between exposure quartile of tepotinib or MSC2571109A and grade ≥ 3 AE, treatment discontinuation due to an AE, and dose reduction due to an AE were also evaluated.

KM plots, boxplots, or spaghetti plots of the safety endpoints, stratified by tepotinib, exposure quartiles were visually inspected to determine the feasibility of a model-based analysis.

#### Time-to-event model for edema

The occurrence of first edema event was described using a TTE model. Exponential (constant hazard), Weibull and Gompertz (hazard changes over time) distributions were tested, and the impact of drug exposure (time-varying AUC_24h_) on the hazard was modeled according to Eq. 1, where h_0_(t) is the base hazard. Covariate (risk factors) on the base hazard were evaluated as shown in Eq. 2, where EFF_drug_ is the drug effect and θ_i_ is the coefficient describing the impact of covariate (risk factor) cov_i_.

1 $$h_{i} \left( t \right) = { }h_{0} \left( t \right) \cdot e^{{{{EFF}}_{{{{drug}}}} }}$$

2 $$h\left( t \right) = { }h_{0} \left( t \right) \cdot { }e^{{\theta_{1} \cdot {{cov}}_{{1{ }}} + \theta_{2} \cdot {cov}_{2} + \cdots + \theta_{n} \cdot {{cov}}_{n} + {{EFF}}_{{{{drug}}}} }}$$

Covariates tested in the edema TTE model were sex, age, body weight, race, tumor type, number of lesions, Eastern Cooperative Oncology Group performance status (ECOG PS) score, metastatic status, number of prior systemic anticancer therapies in the locally advanced/metastatic setting, concomitant diuretic use, and creatinine clearance. The covariate effect was illustrated using Forest plots after 100 bootstraps.

#### Serum albumin model

The time course of the changes in serum albumin was modeled using an indirect response model, assuming zero-order production and first-order degradation. The impact of tepotinib was assumed to affect the zero-order production rate constant (*k*_in_) of albumin. The structural model is described in Eqs. 3 and 4.

3 $$\frac{{{{dAlbumin}}}}{{{{d}}t}} = k_{{{{in}}}} \cdot {{ EFF}}_{{{{drug}}}} - { }k_{{{{out}}}} \cdot {{Albumin}}$$

4 $$k_{{{{in}}}} = {{Albumin}}_{{{{Baseline}}}} \cdot k_{{{{out}}}}$$

*k*_out_ is the first-order degradation rate constant of albumin, and Albumin_Baseline_ is the baseline albumin concentration. Covariates considered in the exposure-serum albumin model were body weight, body mass index, AST, bilirubin, hematocrit, erythrocyte count, hemoglobin, and albuminuria/proteinuria.

#### Drug effect

The exposure metrics for tepotinib and MSC2571109A, used in the graphical analyses and in model-based analysis, were derived from a tepotinib population PK model [14], including individual predictions of longitudinal, time-varying AUC_24h_ (for the edema TTE and albumin modeling), and steady-state AUC (AUC_τ,ss_), AUC_24h_ immediately preceding the event, the time-averaged AUC_24h_ during the week prior to the event, or the time-averaged AUC_24h_ during the 2 weeks prior to the event (for the graphical analyses).

Linear, log-linear, and *E*_max_ (or *I*_max_) models of drug effect (EFF_drug_) were evaluated in the exposure–response models, as indicated by the exploratory graphical analysis (Eqs. 5–8).

5 $${EFF}_{drug} = {Slope}_{drug} \cdot {AUC}_{x}$$

6 $${EFF}_{drug} = {Slope}_{drug} \cdot ln\left( {{AUC}_{x} } \right)$$

7 $${EFF}_{drug} = { }\frac{{E_{\max} { } \cdot { }{AUC}_{x} }}{{{AUC}_{50} + { }{AUC}_{x} }}$$

8 $${EFF}_{drug} = 1 - \frac{{I_{\max} { } \cdot { }{AUC}_{x} }}{{{AUC}_{50} { } + { }{AUC}_{x} }}$$

Slope_drug_ is the slope of the linear drug response relationship, AUC_*x*_ is the individually predicted tepotinib area under the curve exposure metric, *E*_max_ is the maximum effect, *I*_max_ is the maximum inhibition and AUC_50_ is the AUC_*x*_ at half the maximum effect.

#### Covariate modeling

A stepwise covariate model (SCM) building procedure was performed with a forward inclusion phase and backward elimination phase [21]. The forward selection p-value was set to 0.01 and the backward elimination p-value to 0.001. Adaptive scope reduction (ASR) [22] was added to the model to reduce the defined search scope during the forward search (see Supplementary Materials for additional information). Continuous covariate relationships were coded as linear (for logit-transformed parameters) (Eq. 9a) or power models (Eq. 9b), and categorical covariates were coded as a fractional difference to the most common category (Eq. 9c).

9a $${ParCov}_{m} = { }\theta_{m} \cdot \left( {{Cov} -{Cov}_{ref} } \right)$$

9b $${ParCov}_{m} = { }\left( {\frac{{Cov}}{{{Cov}_{ref} }}} \right)^{{\theta_{m} }}$$

9c $${ParCov}_{m} = { }\left\{ {\begin{array}{*{20}l} {1\,\, if\,\, {Cov} = {Cov}_{ref} } \\ {1 + \theta_{m}\,\, if\,\, {Cov} \ne {Cov}_{ref} } \\ \end{array} } \right.$$

where Cov_ref_ is a reference covariate value for covariate m, to which the covariate model is normalized (usually the median or mode).

#### Concentration-QTc analysis

PK time-matched electrocardiograms (ECGs) collected in studies 001, 003, 004, and 005 (tepotinib multiple doses ranging from 30 to 1400 mg) contributed to an integrated concentration-QT interval corrected using Fridericia’s formula (QTcF) analysis [23]. A second concentration-QTcF analysis was performed on centrally read, PK time-matched, triplicate 12-lead digital ECGs collected in cohort A of VISION (tepotinib 500 mg/day). In both analyses, concentrations of tepotinib and its metabolite MSC2571109A were evaluated as the predictor using linear mixed effects models in SAS (v7, Cary, NC). See Supplementary Methods for additional details.

## Results

### Exposure–efficacy analyses

A total of 146 patients with NSCLC harboring *MET*ex14 skipping alterations who received tepotinib 500 mg/day in the pivotal phase 2 VISION study were included in this analysis. The characteristics of patients from the VISION study, according to tepotinib exposure quartile, are shown in Supplementary Table S1. Covariates were generally balanced across tepotinib AUC_τ,ss_ exposure quartiles, with the exception of minor trends towards a lower body weight (< 10% relative difference vs overall mean) and a higher proportion of females (~ 10% difference vs overall mean) with increasing tepotinib exposure quartile. Mean tepotinib AUC_τ,ss_ in the overall population was 25.3 µg·h/mL (range 4.7–51.1 µg·h/mL).

**Fig. 1:**
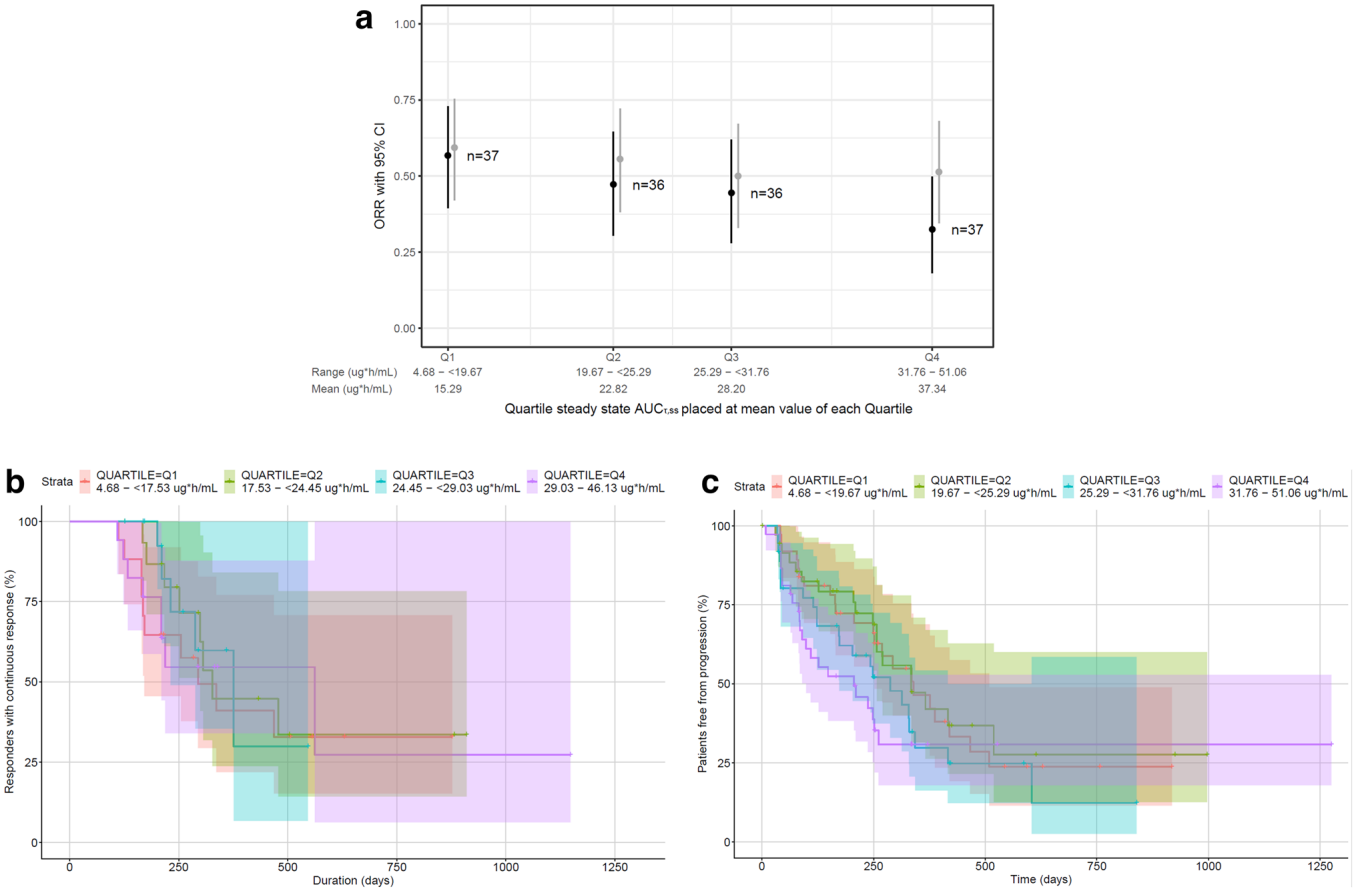
Association between tepotinib exposure and independently assessed (panels **a**–**c**) or investigator-assessed (panel **a**) efficacy outcomes in patients with NSCLC and *MET* exon 14 skipping alterations. Objective response rate (**a**), duration of response (**b**), and progression-free survival (**c**) by tepotinib AUC_τ,ss_ quartile. OR and PFS analyses include all 146 patients; duration of response is based on 66 patients who attained an objective response. The lines represent the Clopper–Pearson 95% CI and points are observed OR per AUC quartile (dark gray represents OR assessed by independent evaluation, and light gray represents OR assessed by investigator review). In panels **b** and **c**, shaded areas represent 95% confidence intervals. *AUC*_*τ,ss*_, area under the curve at steady state; *CI*, confidence interval; *NSCLC*, non-small cell lung cancer; *OR*, objective response; *ORR*, objective response rate; *PFS*, progression-free survival

All 146 patients were included in the exposure–efficacy analyses for OR and PFS, and 66 patients who attained a response were included in exposure–efficacy analyses for DOR. Graphical analysis indicated that increasing tepotinib exposure was not associated with higher OR according to investigator- and independent-assessments, with OR 95% CIs that overlapped across exposure quartiles (Fig. 1a). Increasing tepotinib exposures were also not associated with DOR or PFS as assessed by independent evaluation (Fig. 1b–c), or by investigator assessment (data not shown). There was no clear association between OR, DOR or PFS, and the covariates (race, sex, ECOG PS, line of therapy, presence of central nervous system metastases, *MET*ex14 skipping alteration diagnosis [tumor vs liquid biopsy], histology [adenocarcinoma vs non-adenocarcinoma], body weight, number of non-target lesions at baseline or sum of longest diameters for target lesions at baseline).

### Exposure–safety analyses

A total of 499 patients from five clinical trials who received multiple doses of tepotinib monotherapy ranging from 30 to 1400 mg/day were included in the exposure–safety analyses (Supplementary Table S2). Most patients were male (63.1%) and either Caucasian (57.1%) or Asian (29.9%) race. Median age was 66 years (range 19–93 years). NSCLC (40.3%) and HCC (27.5%) were the common tumor types, 68.9% had an ECOG PS of 1, and metastatic disease was present in 98.6% of patients.

#### Edema and serum albumin

**Fig. 2:**
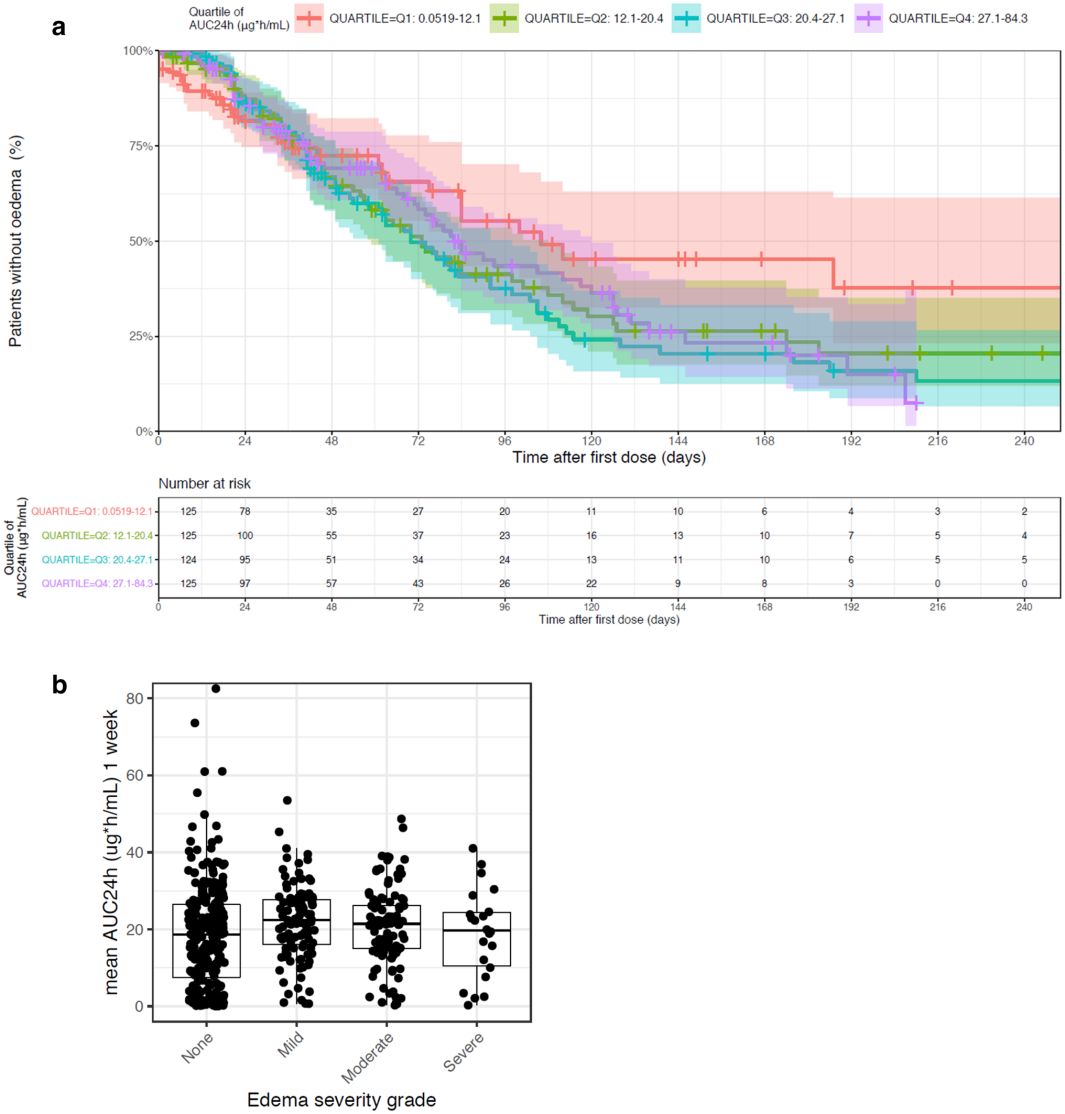

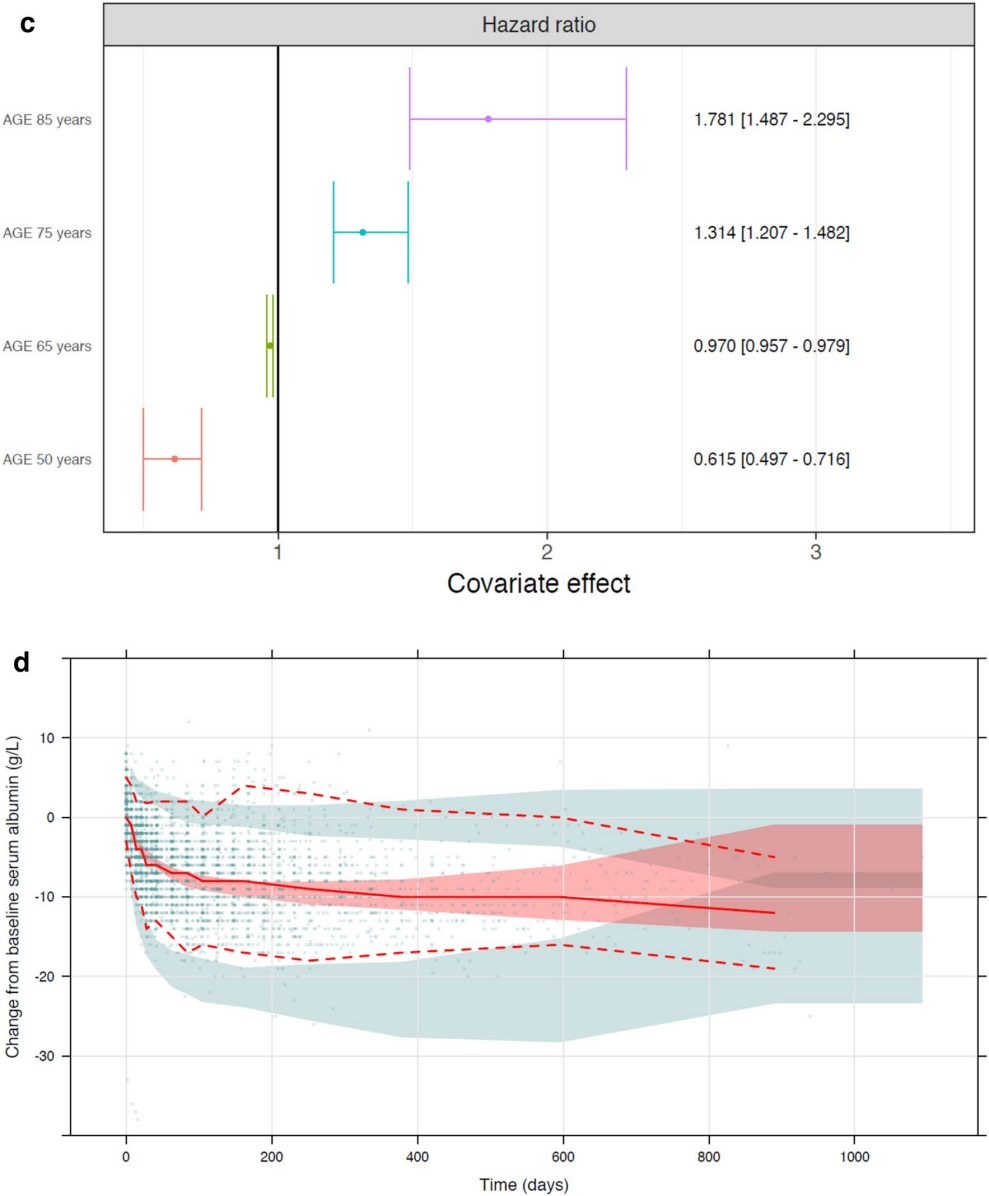

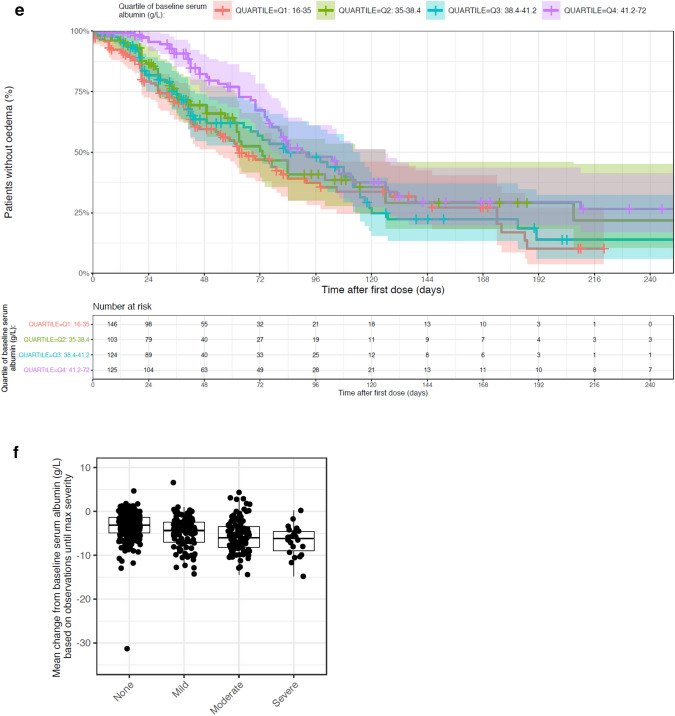
Relationship between tepotinib AUC_24h_ quartile and edema events and change in serum albumin levels. Panel **a** presents time-to-first edema event stratified by tepotinib AUC_24h_ quartile on the day of the edema event or day of censoring. Panel **b** presents the distribution of mean tepotinib AUC_24h_ during 1 week prior to an edema event according to edema severity (maximum severity per participant). Panel **c** presents impact of age on the predicted risk of edema based on the final TTE model with model-estimated hazard ratios for edema relative to a typical participant of median age of 66 years (the closed symbols represent the median hazard ratio for the applicable age category. The whiskers represent the 90% CI of the median values, based on 100 bootstrap datasets. The vertical black line represents the hazard ratio for a typical patient in the analysis data set, aged 66 years). Panel **d** presents the visual predictive check of the indirect response model of serum albumin with an inhibitory effect of tepotinib exposure on albumin formation. In panels **a** and **d**, shaded areas represent 95% CI. In panel **e**, solid and dashed red lines represent the observed median, 5th and 95th percentiles; the shaded red area represents the 95% CI of the model predicted median, and the shaded blue areas represent the 95% CI of the model predicted 5th and 95th percentiles. Dots are observed values. Panel **e** presents a Kaplan–Meier analysis of time-to-first edema event stratified by quartiles of baseline serum albumin. Panel **f** presents mean change from baseline serum albumin according to edema severity. *AUC*_*24h*_, 24-h area under the curve; *CI*, confidence interval; *TTE*, time-to-event

Of the 499 patients in the pooled safety analysis set, 239 patients (47.9%) had at least one edema event. KM analysis of edema incidence indicates a longer time-to-first edema event within the lowest tepotinib AUC_24h_ quartile (0.05–12.1 µg·h/mL) relative to tepotinib AUC_24h_ > 12.1 μg·h/mL (i.e., quartiles 2–4) (Fig. 2a). The distribution of tepotinib exposure (defined as mean AUC_24h_ during the week prior the edema event) was similar across all edema severity grades (Fig. 2b). This observation also remained consistent when mean tepotinib AUC_24h_ up to the time of the event and mean tepotinib AUC_24h_, during the 2 weeks prior to the edema event, were employed as metrics of tepotinib exposure (data not shown).

A model-based evaluation of the relationship between tepotinib exposure and the first occurrence of edema was performed using a TTE model (Supplementary Table S3). A constant hazard (exponential distribution) was found to provide the best description of the base hazard. Tepotinib exposure, expressed as time-varying AUC_24h_, did not have a discernible impact on the hazard model with a drop in objective function value (OFV) of less than –1.6 for all tested exposure–response models. A visual predictive check of the base model confirmed that it adequately described the probability of edema during tepotinib treatment (Supplementary Fig. S1). The final TTE model also revealed that advanced age was associated with an increased risk of edema, independent of tepotinib exposure. The median hazard ratio for risk of edema was estimated to be 1.3 (90% CI 1.2, 1.5) for a 75-year-old patient relative to a typical reference 66-year-old patient (median age in the analysis population) (Fig. 2c). There was no discernible association between any other variables and risk of edema (a full list of the variables included in the TTE model is provided in Supplementary Table S2).

Median baseline serum albumin concentration was 38.4 g/L (range 16.0–72.0 g/L). There was a trend toward decreasing serum albumin concentrations with time in all studies. The time course of serum albumin concentration was described using an indirect response model, with tepotinib exposure-related inhibition of the formation of albumin (i.e. exposure-related decrease in the formation rate constant k_in_ in a Michaelis–Menten fashion). The visual predictive check plot of the indirect response model across the full population is shown in Fig. 2d. The model estimated a 26.1% (relative standard error [RSE] 2.21%) decrease in serum albumin at steady state, which is reached approximately on Day 133. The AUC_50_ associated with this effect was estimated to be 0.215 μg·h/mL (RSE 24.5%) (Supplementary Table S4). This represents 1% of the AUC_τ,ss_ at clinical dose, suggesting that the time course of the effect on albumin was likely driven by the turnover rate of albumin, rather than an accumulation of tepotinib exposure.

The association between change in serum albumin levels and risk of edema was graphically evaluated. The risk of edema within 72 days of initiating treatment appeared to be slightly lower in patients within the highest quartile of baseline serum albumin (> 41 g/L) (Fig. 2e). However, there was no clear association between the time to the first edema event and the mean serum albumin concentrations (data not shown). The magnitude of decrease in serum albumin over time also appeared to be positively associated with maximum severity of edema (Fig. 2f). This trend was apparent when change in serum albumin was assessed, based on all observations up to the time of the most severe edema events (as shown in Fig. 2f), and when change in serum albumin was based on all reported serum albumin observations (data not shown). However, there was no clear association between baseline albumin concentration and the severity of edema. It is important to note that there was substantial variability in serum albumin levels at baseline and that approximately 25% of patients had baseline albumin concentrations that were lower than the lower limit of normal range.

#### Serum creatinine

**Fig. 3:**
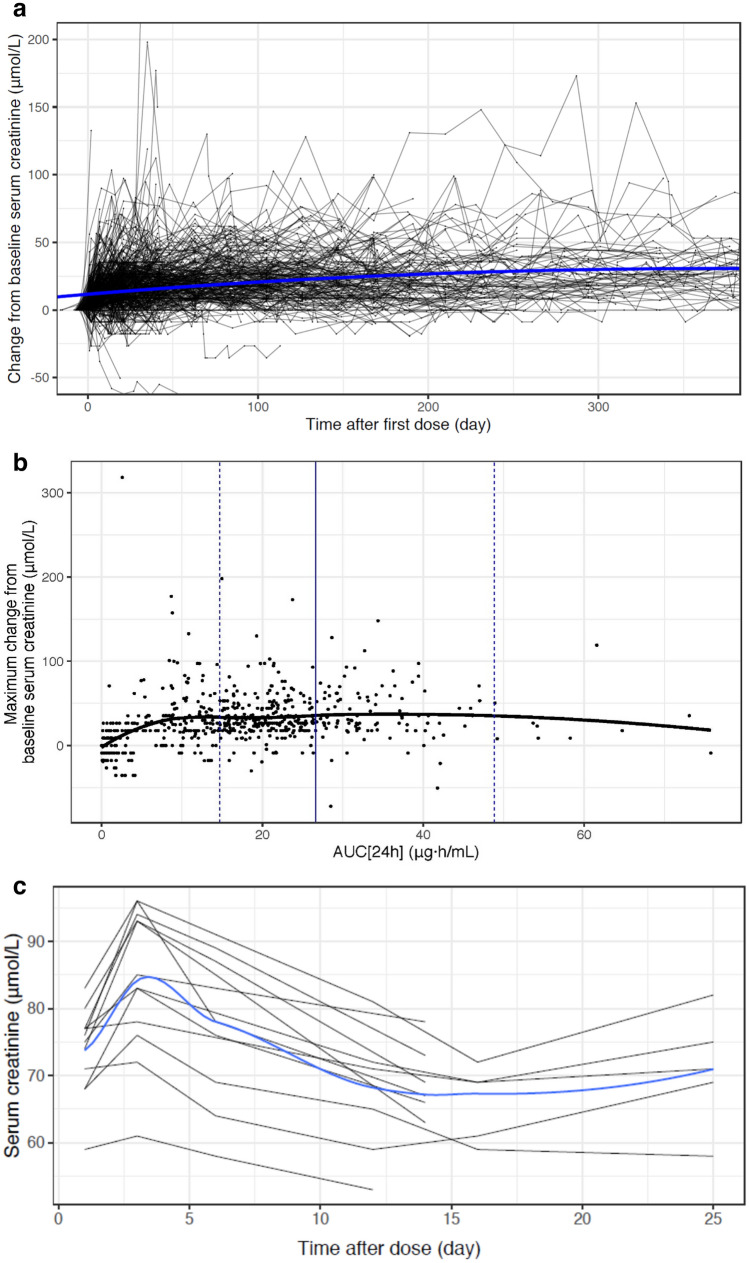
Change in serum creatinine following tepotinib administration. Panel **a** presents the change from baseline in serum creatinine concentrations following first dose of study medication for all individual patients. Each line represents the data for one participant. The solid blue line is a LOESS smooth. The y-axis is truncated at -50 and 200 μmol/L and the x-axis at 365 days. Panel **b** presents the individual maximum change from baseline in serum creatinine concentration versus tepotinib AUC_24h_. Dots represent observations. The solid black line is a LOESS smooth. The vertical blue lines indicate the PK model simulated median (solid line), 5^th^ and 95^th^ percentiles (dashed lines) of AUC_τ,ss_ at a dose of 500 mg. Panel **c** presents individual serum creatinine concentrations taken from 56 observations from 11 patients over time following a single administration of tepotinib (500 mg) in healthy volunteers in study 007. The black lines represent individual patient data and the blue line is a LOESS smooth. *AUC*_*24h*_, 24-h area under the curve; *AUC*_*τ,ss*_, area under the curve at steady state; *LOESS,* locally estimated scatterplot smoothing; *PK*, pharmacokinetics

Graphical analysis indicates a consistent trend of increasing serum creatinine concentration over time which reached a plateau with continued tepotinib exposure (Fig. 3a). The maximum increase in serum creatinine was on average approximately 30 μmol/L and appeared to saturate at a tepotinib AUC_24h_ of approximately 10 μg·h/mL (representing 45% of the tepotinib AUC_τ,ss_ at clinical dose), or an MSC2571109A AUC_24h_ of approximately 5 μg·h/mL (representing 66% of the metabolite AUC_τ,ss_ at clinical dose) (Fig. 3b and Supplementary Fig. S2). To explore the reversibility of the increase in serum creatinine following tepotinib administration, serial-sampled serum creatinine levels were assessed following a single dose administration of tepotinib in healthy volunteers (Fig. 3c). The time course suggests a rapid reversal of serum creatinine changes, returning toward baseline concentrations approximately 10 h following a single dose.

#### QTc interval

**Fig. 4:**
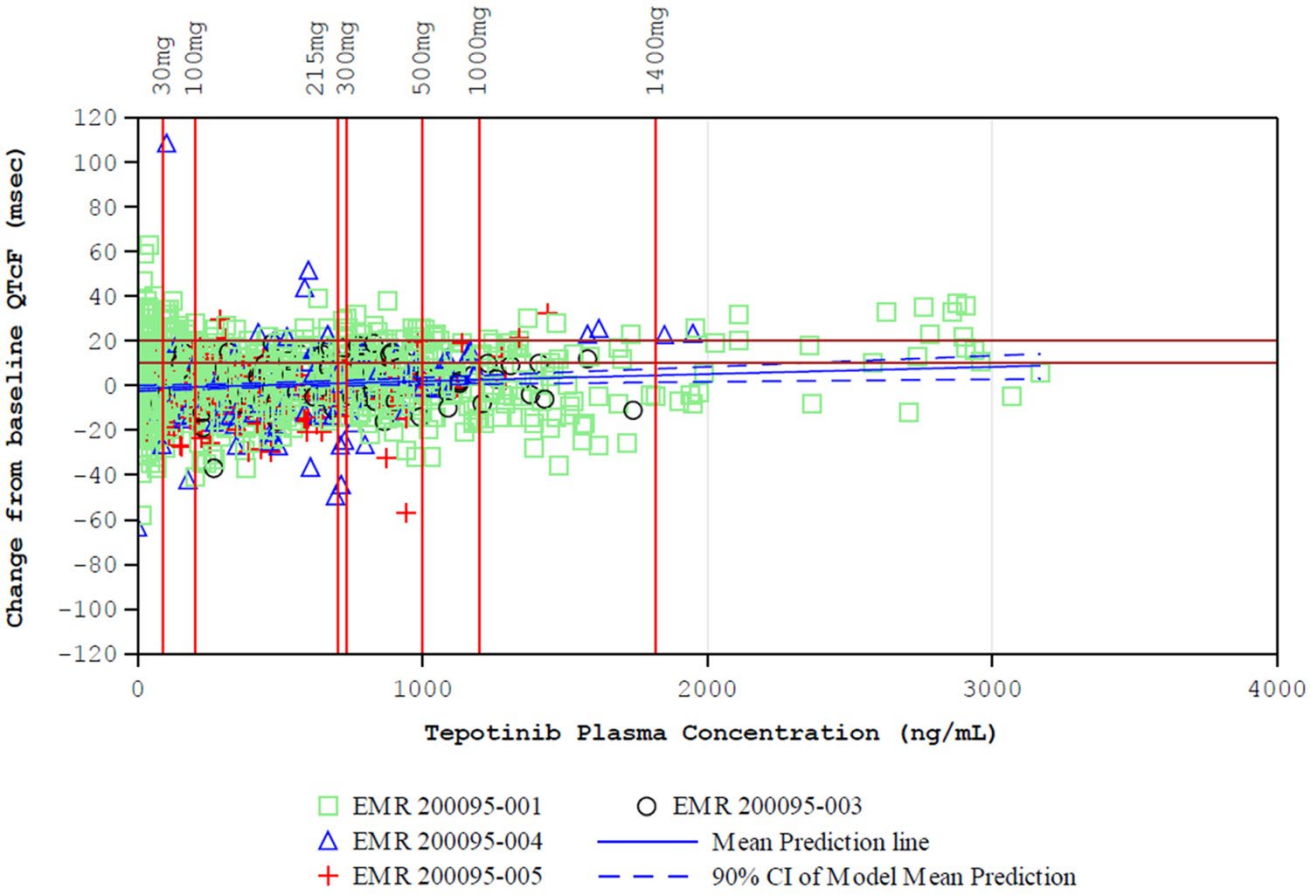
Relationship of ΔQTcF interval versus tepotinib plasma concentration. The model derived predicted population ΔQTcF from baseline is shown as the continuous blue line and the two-sided 90% bootstrapped confidence limits of predicted mean ΔQTcF are shown as broken lines for pooled study patients. The vertical red lines correspond to geometric mean C_max_ at steady state in the 500 mg and 1400 mg dose levels. The brown horizontal lines represent the regulatory threshold of potential concern of 10 ms, and an additional 20 ms reference line as a threshold of potential clinical relevance applicable for oncology drugs. Open symbols represent observed data. *CI*, confidence interval; *QTcF*, QT interval corrected using Fredericia’s formula

Linear mixed effects modeling was used to quantitatively assess the effect of tepotinib concentration on QTcF. The model-based regression line of the population mean ΔQTcF and its two-sided 90% CIs, obtained by bootstrapping of 1000 datasets, is shown in Fig. 4. There was a slight increase in ΔQTcF with increasing tepotinib exposure. The upper bound of the 90% CIs of the predicted mean ΔQTcF were 3.57 ms at the observed geometric mean steady-state *C*_max_ at the proposed clinical dose of 500 mg, and 7.54 ms at the geometric mean steady-state *C*_max_ at the highest administered dose of 1400 mg (Supplementary Table S5). The impact of MCS2571109A was assessed in patients for whom matched tepotinib and MCS2571109A concentrations and ΔQTcF data were available using multivariate regression. At the proposed tepotinib clinical dose of 500 mg, the mean predicted ΔQTcF was 3.1 ms at a tepotinib *C*_max_ of 1000.2 ng/mL and MSC2571109A *C*_max_ of 319.3 ng/mL. At a tepotinib dose of 1000 mg, the mean predicted ΔQTcF was 5.2 ms at a tepotinib *C*_max_ of 1199.4 ng/mL and MSC2571109A *C*_max_ of 384.4 ng/mL. The upper bound of the 90% CIs of the predicted mean ΔQTcF at the observed geometric mean steady-state *C*_max_ was 4.3 ms at the proposed clinical dose of 500 mg, and 6.8 ms at the highest administered dose of 1000 mg.

Similarly, an additional QTc analysis of tepotinib at the clinical dose of 500 mg in cohort A of the VISION study (*N* = 107 patients) showed that the upper bound of the 90% confidence interval of the estimated population mean ΔQTcF was 7.9 ms (Supplementary Table S5). Similar results were obtained in VISION cohort A with matched tepotinib and MCS2571109A concentrations; timepoint and categorical analyses for both the integrated population and the VISION cohort did not show any clinically significant changes (Supplementary Tables S6–9).

#### Lipase, amylase, ALT, and AST

Trends towards treatment-emergent amylase increase, and transient increases in AST and ALT were noted. There was no discernible association between tepotinib exposure and median observed increases, or median relative change from baseline for lipase, amylase, ALT or AST (data not shown).

#### Severe AEs/dose reductions or treatment interruption due to AEs

In updated safety analyses of the VISION study, comprising all patients in cohorts A and C who received tepotinib by July 1, 2020 (*N* = 255), treatment-related AEs led to dose reductions in 71 patients (27.8%), to temporary treatment discontinuations in 90 patients (35.3%), and to permanent treatment discontinuation in 27 patients (10.6%) [24]. The median dose intensity corresponds to 99.6% of the target dose intensity.

**Fig. 5:**
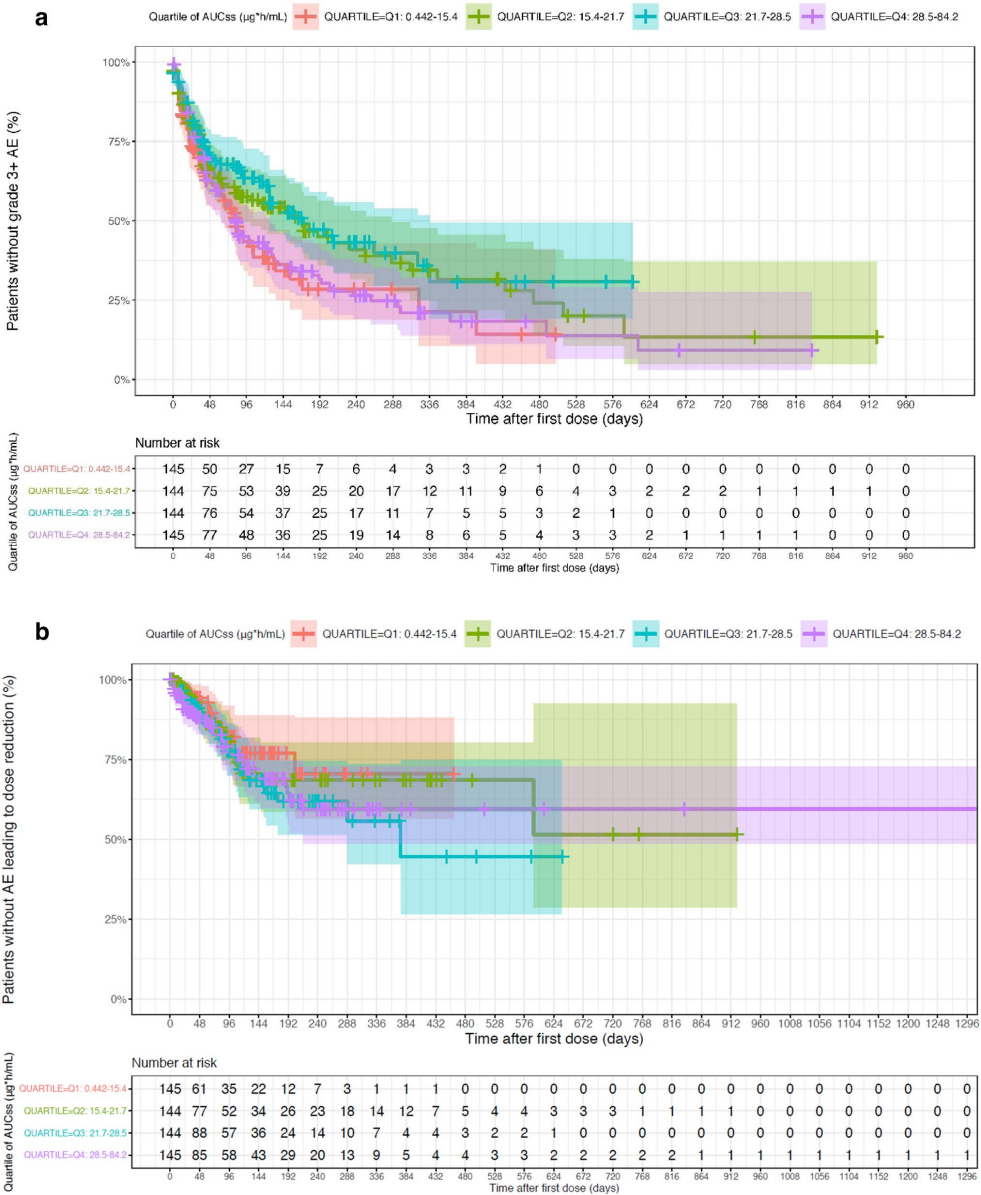
Relationship between tepotinib exposure and grade ≥ 3 adverse event, and dose reduction due to an adverse event. Panel **a** presents Kaplan–Meier analysis of time-to-first grade ≥ 3 AE stratified according to tepotinib exposure quartile. Panel **b** presents Kaplan–Meier analysis of time-to-first dose reduction due to an AE stratified according to tepotinib exposure quartile. Shaded areas represent 95% confidence intervals. *AE*, adverse event; *AUC*_*τ,ss*_, area under the curve at steady state

There was no clear association between tepotinib or MSC2571109A AUC_τ,ss_ and grade ≥ 3 AE or dose reduction, treatment interruption, or permanent treatment discontinuation due to an AE (Fig. 5 and Supplementary Figs. S3 and S4). Furthermore, there is no indication that exposure to MSC2571109A is a more accurate predictor of safety endpoints than tepotinib exposure.

## Discussion

Rational dose selection and pharmacologic contextualization of the benefit/risk profile of the recommended clinical dosage, including dose modifications for treatment-emergent toxicities, is a critical component of anticancer drug development [25, 26]. This is particularly crucial in the development of molecularly targeted agents, where dosing at or near the maximum tolerated dose without appropriate pharmacologic contextualization, can compromise the overall benefit/risk profile due to poor long-term tolerability [27, 28]. This raises important opportunities for PD biomarker and PK/PD model-informed approaches to rational dose selection [29, 30]. Tepotinib, a highly selective inhibitor of the MET receptor tyrosine kinase, was developed using a fully biomarker-driven and model-informed approach to dose selection in early development, with the recommended phase 2 dose of 500 mg/day selected to provide sustained maximal target inhibition in tumor tissue, based on integrated modeling of preclinical PK/PD relationships, clinical PK, and tumor PD data evaluating inhibition of tumor MET phosphorylation in the first-in-human study [14, 31]. Efficacy and overall benefit/risk of the 500 mg/day dosage for the treatment of NSCLC harboring *MET*ex14 skipping alterations have been demonstrated in the pivotal phase 2 VISION trial [6]. Herein, we report exposure–response analyses of the efficacy of tepotinib in the VISION trial in patients with NSCLC with *MET*ex14 skipping alterations, and integrated exposure–safety analyses for key safety/tolerability outcomes across multiple clinical studies of tepotinib, aimed at quantitative pharmacologic contextualization of the benefit/risk profile of the recommended clinical dosage.

### Clinical efficacy

Graphical and model-based analyses suggest a flat exposure–efficacy relationship for OR, DOR, and PFS in the VISION study. It is in agreement with our dose selection rationale that 500 mg/day regimen of tepotinib is expected to achieve close-to-complete (≥ 95%) intra-tumoral phospho-MET inhibition in the majority (> 90%) of treated patients [14], and renders clinical efficacy independent of individual factors that may influence exposure. At a reduced tepotinib dose of 250 mg/day, which is recommended to manage AEs, targeted sustained nearly-complete MET inhibition (≥ 95%) would still be expected in ≥ 80% of patients. The 90% prediction interval of AUC_τ,ss_ at the 250 mg/day (8.1–26.9 µg·h/mL) falls within the observed tepotinib AUC range achieved in the VISION study, in which a flat exposure–efficacy relationship was observed. These data, therefore, indicate that efficacy would be maintained in patients who require a temporary dose reduction to 250 mg/day for the management of AEs. This is also supported by the observation that patients, with dose reductions in the VISION study, remained on treatment and continued to benefit from tepotinib for prolonged periods [32]. However, the majority of AEs reported in patients receiving tepotinib in clinical trials to date did not require dose modification.

### Edema

The most frequently reported AE for tepotinib is edema. While graphical analyses indicated that the risk of edema appeared to be lower in patients with tepotinib exposures within the lowest quartile, no readily apparent association between tepotinib exposure and edema grade was observed, and model-based analysis did not identify a discernible relationship between tepotinib exposure and the risk of edema. In summary, development of edema is clearly associated with the administration of tepotinib, but the effect seemed to plateau at low tepotinib exposures and the underlying exposure–response relationship therefore could not be fully quantified in the present analyses. Edema was also the most frequent cause of dose reductions and treatment interruptions in the VISION study, with a median time-to-first onset of 7.9 weeks (range 0.1–58.3) [6, 24]. The present TTE model indicated that advanced age was associated with an increased risk of edema, independent of tepotinib exposure, and consistent with an age of > 70 years typically seen in patients with *MET*ex14 skipping alterations [33].

Edema is a commonly reported AE among patients receiving MET inhibitors [34–36], suggesting that the underlying pathology is possibly a target-mediated effect. Some evidence points to a role for the MET/PI3k/Akt pathway and the MET ligand, HGF, in the modulation of endothelial permeability [37, 38]. Inhibition of the HGF/MET signaling axis may, therefore, lead to a reduction in the integrity of the endothelial barrier and subsequent fluid accumulation and edema.

Therefore, it is not surprising that both clinical efficacy and development of edema follow the same flat exposure–response relationship in this MET-driven tumor indication. This also suggests that temporary treatment interruptions, rather than dose reduction, may be a more effective approach to managing patients who develop edema.

### Serum albumin

A trend towards decreasing serum albumin concentrations over time was noted in patients receiving tepotinib, with a decrease of 26% at steady state. Model-based analysis suggests that the inhibitory effect saturates at low exposure of tepotinib. Furthermore, both the risk and severity of edema were associated with serum albumin, with high baseline albumin likely providing some protection against early development of edema. There was also an apparent trend for positive association between magnitude of decrease in serum albumin and maximum severity of edema, with more severe edema seen in patients with the greatest reduction in serum albumin. This highlights an opportunity for further analyses to quantitatively evaluate the link between time course of changes in albumin and time course of edema. However, both the risk of edema and effect on serum albumin appeared to plateau at low exposure of tepotinib.

The underlying mechanism(s) are poorly understood. Treatment-emergent hypoalbuminemia was also observed in other MET inhibitors [39, 40]. Owing to its physiologic role of maintaining oncotic pressure, decreases in serum albumin may be an independent factor for edema pathogenesis.

### Serum creatinine

Tepotinib treatment was associated with, on average, an approximately 30 μmol/L maximum increase in serum creatinine levels. This increase in serum creatinine plateaued with time and continued drug exposure, and was found to be reversible, based on data from healthy volunteers receiving single dose administration. No other clinical laboratory or clinical findings suggested a relation to kidney injury.

A potential explanation for the increase in serum creatinine is that tepotinib or MSC2571109A inhibit the elimination of creatinine through inhibition of the organic cation transporter 2 (OCT2) or the multidrug and toxin extrusion (MATE) transporters. At clinical doses, tepotinib reaches a steady-state free peak plasma concentration of 0.05 μM whilst inhibiting MATE1 with an IC_50_ of 3.6 μM and MATE2 with an IC_50_ of 1.1 μM, and MSC2571109A reaches a steady-state free peak concentration of 0.01 μM whilst inhibiting OCT2 with an IC_50_ of 0.04 μM.

This hypothesis is supported by a recent report from Mathialagan and colleagues [41], who found that serum creatinine can be increased by inhibition of renal transporters, including OCT2 and MATE1, without renal toxicity. Furthermore, a case report from Mohan and Herrmann showed that, despite elevations in serum creatinine levels after treatment with the MET inhibitor capmatinib, estimated glomerular filtration rate (eGFR) derived from cystatin C and renal iothalamate clearance was stable [42]. OCT2 and MATE1/2 inhibition may also contribute to treatment-emergent transient increases in creatinine with tucatinib [43], and can be described using physiologic modeling [44]. From a practical standpoint, the available data with tepotinib suggest that renal function markers that rely solely on serum creatinine levels (creatinine clearance, eGFR) should be treated with some caution when measured during tepotinib pharmacotherapy, and that careful consideration should be given prior to basing dose adjustment recommendations on such data. Based on the absence of clinical signs or other lab markers of renal toxicity, e.g., electrolytes, urea, the observed increases in creatinine have no causal relationship to edema.

### QTc interval

Concentration–QTc analyses for tepotinib and MSC2571109A showed no evidence of a clinically significant prolongation effect on QTcF interval. Based on linear mixed effects modeling, QTc prolongation did not exceed the threshold of 10 ms for either the proposed clinical dose of 500 mg, or for the highest administered dose of 1400 mg. In vivo and in vitro safety pharmacology data also suggest no anticipated risk for QT prolongation at the clinical dose of tepotinib (data on file). In these studies, tepotinib inhibited Kv11.1 (hERG) with an IC_50_ of 1.2 µM, which is 24-fold higher than the mean unbound steady-state *C*_max_ of 0.05 µM achieved with the 500 mg clinical dose. There was also no meaningful effect of tepotinib on other key cardiac ion channels (hNav1.5, hKv1.5, hKv4.3/hKChIP2, Cav1.2, hKCNQ1/hminK, hHCN4, and hKir2.1) up to the highest tested concentration of 10 µM. MSC2571109A had no effect on key cardiac ion channels, and no effect of tepotinib, or MSC2571109A was seen in dedicated cardiovascular safety pharmacology studies in rats and dogs.

The present exposure–safety analysis included a large number of patients pooled from different phase 1/2 trials, providing a robust assessment of the underlying exposure–response relationships. The flat exposure–efficacy relationship is consistent with the quantitative understanding of target modulation and, therefore, represents a classic case study of a model-informed dosing strategy confirmed by clinical data. A limitation of this analysis is that clinical situation with edema varies in anatomic location and intensity, as well as mixed and combined countermeasures, including overlapping dose reductions and temporary treatment interruptions plus other supportive therapies. The longitudinal profiles of edema, including onset/offset and severity of each episode, and its response to dose reduction/treatment interruption are still under investigation. The association between edema and serum albumin decrease has been observed in the exploratory graphical analysis, and further model-based characterization may provide some insight to support the hypothesis of its causal relationship. Such data will be necessary to inform the development of more comprehensive, pharmacometric models that may help elucidate the complex inter-relationships between the time course of tepotinib exposure, serum albumin, and the onset, severity and offset of edema.

In conclusion, a flat exposure–efficacy relationship was observed within the exposure range achieved after administration of tepotinib 500 mg/day in patients with advanced NSCLC harboring *MET*ex14 skipping alterations. The relationships between tepotinib exposure and edema, serum albumin, creatinine, lipase, amylase, AST, and ALT were also flat within the observed exposure range at a 500 mg daily dose. Concentration-QTc analyses indicate that tepotinib does not produce clinically relevant increases in the QTcF interval at the 500 mg daily dose. Taken together, these exposure–response analyses provide important quantitative pharmacologic support for benefit/risk assessment of the 500 mg once daily dosage of tepotinib, as being appropriate for the treatment of NSCLC harboring *MET*ex14 skipping alterations.

## Supplementary Information

Below is the link to the electronic supplementary material.

Supplementary file1 (DOCX 843 kb)

## Declarations

### Data availability statement

Any requests for data by qualified scientific and medical researchers for legitimate research purposes will be subject to Merck KGaA’s Data Sharing Policy. All requests should be submitted in writing to Merck KGaA’s data sharing portal (https://www.merckgroup.com/en/research/our-approach-to-research-and-development/healthcare/clinical-trials/commitment-responsible-data-sharing.html).
