# Supplementary material for: Exposure–response analyses for the MET inhibitor tepotinib including patients in the pivotal VISION trial: support for dosage recommendations
Source: Cancer Chemother Pharmacol. 2022 Jun 30;90(1):53–69. doi: 10.1007/s00280-022-04441-3 (PMC9300558; doi:10.1007/s00280-022-04441-3)
Supplement: Supplementary file 1 — Supplementary file1 (DOCX 843 kb) [file 280_2022_4441_MOESM1_ESM.docx]

# Supplemental Materials

### Covariate Modeling Adaptive Scope Reduction (ASR)

With ASR, the first forward step was identical to the standard stepwise covariate model algorithm but in the second step, any covariate-parameter relationship that was less significant than the prespecified threshold of *p*=0.01 was removed from the search scope before the most significant covariate-parameter relationship was identified for retention. This process was repeated for each subsequent forward step until no additional covariate parameter relationship could be added to the model. The forward search(es) were then followed by a backward elimination.

### QTc interval

An integrated concentration-QTc (cQTc) analysis was performed using electrocardiogram (ECG) data, with time-matched tepotinib and MSC2571109A plasma concentration datasets from studies 001, 003, 004, and 005 studies (tepotinib single or multiple dose ranging from 30 mg to 1,400 mg). Between-study homogeneity was assessed by estimating the slopes of effects for each individual study, using study and study*concentration as additional effects to confirm validity of pooling.

Concentration-QTc analyses were also conducted using ECG data from VISION study (tepotinib starting dose 500 mg/day). Serial pharmacokinetic (PK) time-matched 12-lead digital ECGs were recorded in triplicate on Cycle 1, Day 1 and on Cycle 2, Day 1, predose (within 30 minutes prior to dose) and at 4 hours post-dose. To ensure high-quality ECG data, the ECGs recorded during Cycle 1 and 2 of the study were assessed at a central ECG laboratory by expert readers who were blinded to treatment allocation and timepoint using the QECGBuild system (IQVIA, Cardiac Safety Services and Connected Devices).

Linear mixed effects modeling (SAS, Cary, NC) was used to quantify the relationship between tepotinib and MSC2571109A plasma concentrations and ΔQTcF, with baseline QTcF and tepotinib concentration as fixed effects, and slope and intercept for concentration of tepotinib as a participant specific random effect. In addition, a multivariate linear mixed effect model with baseline, tepotinib concentration, MSC2571109A concentration, and the interaction term between tepotinib and MSC2571109A concentration was used for evaluating the effect of both tepotinib and its metabolite on QTcF.

### Model evaluation

The discrimination between models was made based on the inspection of graphical diagnostics, changes in the objective function value (OFV), provided by NONMEM (*p*<0.05) for hierarchical models or AIC criterion for non-hierarchical models, and plausibility of parameter estimates with reasonable relative standard errors (RSEs).

### Additional results on concentration-QTc analysis

In the integrated analysis across studies 001, 003, 004, and 005 studies (*N=*285 patients), three patients with QTc shortening >3 standard deviations (~75 msec) from the mean were excluded following homogeneity analysis. Upon exclusion of these three patients, the slopes of effects for either tepotinib or MSC2571109A for all studies were comparable.

**Supplementary Table S1** Demographics of patients included in exposure-efficacy analysis from the VISION study stratified by tepotinib AUC_τ,ss_ quartile

|  | First quartile (*N=*37) | Second quartile (*N=*36) | Third quartile (*N=*36) | Fourth quartile (*N=*37) | All patients (*N=*146) |
| --- | --- | --- | --- | --- | --- |
| Sex, n (%) |  |  |  |  |  |
| Male | 23 (62.2) | 21 (58.3) | 16 (44.4) | 16 (43.2) | 76 (52.1) |
| Female | 14 (37.8) | 15 (41.7) | 20 (55.6) | 21 (56.8) | 70 (47.9) |
| Race, n (%) |  |  |  |  |  |
| Caucasian | 30 (83.3) | 29 (80.6) | 23 (65.7) | 20 (55.6) | 102 (71.3) |
| Black | 0 (0) | 0 (0) | 0 (0) | 1 (2.8) | 1 (0.7) |
| Asian | 5 (13.9) | 7 (19.4) | 11 (31.4) | 15 (41.7) | 38 (26.6) |
| Hispanic | 0 (0) | 0 (0) | 1 (2.9) | 0 (0) | 1 (0.7) |
| Other | 1 (2.8) | 0 (0) | 0 (0) | 0 (0) | 1 (0.7) |
| Missing | 1 | 0 | 1 | 1 | 3 |
| Body weight, mean (SD), kg | 72.0 (16.3) | 69.4 (12.1) | 61.6 (12.4) | 59.9 (12.2) | 65.8 (14.2) |
| Line of therapy, n (%) |  |  |  |  |  |
| First | 17 (45.9) | 17 (47.2) | 16 (44.4) | 15 (40.5) | 65 (44.5) |
| Second | 11 (29.7) | 13 (36.1) | 13 (36.1) | 10 (27.0) | 47 (32.2) |
| Third | 9 (24.3) | 6 (16.7) | 7 (19.4) | 12 (32.4) | 34 (23.3) |
| Histology, n (%) |  |  |  |  |  |
| Adenocarcinoma | 35 (94.6) | 28 (77.8) | 33 (91.7) | 31 (83.8) | 127 (87.0) |
| Non-adenocarcinoma | 2 (5.4) | 8 (22.2) | 3 (8.3) | 6 (16.2) | 19 (13.0) |
| ECOG PS, n (%) |  |  |  |  |  |
| 0 | 7 (18.9) | 12 (33.3) | 10 (27.8) | 8 (21.6) | 37 (25.3) |
| 1 | 30 (81.1) | 24 (66.7) | 26 (72.2) | 29 (78.4) | 109 (74.7) |
| Non-target lesions, mean (SD) | 2.4 (1.2) | 1.9 (1.3) | 2.4 (1.2) | 2.6 (1.5) | 2.3 (1.3) |
| Sum of longest diameter for target lesions, mm, mean (SD) | 67.8 (40.3) | 50.7 (28.2) | 63.7 (29.8) | 67.7 (45.4) | 62.6 (37.0) |
| *MET* exon 14 skipping alteration detection |  |  |  |  |  |
| LBx+, TBx+ | 10 (27.0) | 6 (16.7) | 10 (27.8) | 7 (18.9) | 33 (22.6) |
| LBx+, TBx- | 2 (5.4) | 0 (0) | 1 (2.8) | 1 (2.7) | 4 (2.7) |
| LBx-, TBx+ | 11 (29.7) | 13 (36.1) | 10 (27.8) | 9 (24.3) | 43 (29.5) |
| LBx+, TBx N/A | 12 (32.4) | 14 (38.9) | 13 (36.1) | 19 (51.4) | 58 (39.7) |
| LBx N/A, TBx+ | 2 (5.4) | 3 (8.3) | 2 (5.6) | 1 (2.7) | 8 (5.5) |
| CNS metastases |  |  |  |  |  |
| No | 33 (89.2) | 34 (94.4) | 32 (88.9) | 33 (89.2) | 132 (90.4) |
| Yes | 4 (10.8) | 2 (5.6) | 4 (11.1) | 4 (10.8) | 14 (9.6) |

*AUC_τ,ss_*, area under the curve at steady state; *CNS*, central nervous system; *ECOG PS*, Eastern Cooperative Oncology Group performance status; *LBx*, liquid biopsy; *SD*, standard deviation; *TBx*, tumor biopsy.

**Supplementary Table S2** Baseline continuous and categorical covariate statistics for patients in the safety analysis set (*N=*499)

|  | Safety analysis set (*N=*499) |
| --- | --- |
| Sex, n (%) |  |
| Male | 315 (63.1) |
| Female | 184 (36.9) |
| Age, median (range), years | 66.0 (19.0, 93.0) |
| Race, n (%) |  |
| Missing | 24 (4.8) |
| Caucasian | 285 (57.1) |
| African origin | 10 (2.0) |
| Asian | 149 (29.9) |
| Hispanic | 20 (4.0) |
| Other/unknown | 11 (2.2) |
| Body weight, median (range), kg | 68.6 (35.5, 139) |
| Body mass index, median (range), kg/m^2^ | 24.5 (13.9, 48.2) |
| Tumor type, n (%) |  |
| HCC | 137 (27.5) |
| NSCLC | 201 (40.3) |
| Other solid tumors | 161 (32.3) |
| ECOG PS, n (%) |  |
| 0 | 155 (31.1) |
| 1 | 344 (68.9) |
| Metastatic disease, n (%) |  |
| No | 7 (1.4) |
| Yes | 492 (98.6) |
| Number of lesions, median (range) | 8.00 (0, 32.0) |
| Prior anti-cancer therapies, n (%) |  |
| Missing | 89 (17.8) |
| 1 | 35 (7.0) |
| ≥2 | 375 (75.2) |
| Urine protein levels, n (%) |  |
| Normal | 279 (55.9) |
| Not normal | 159 (31.9) |
| Missing | 61 (12.2) |
| Bilirubin, median (range), µmol/L | 9.00 (2.00, 48.0) |
| Serum albumin, median (range), g/L | 38.4 (16.0, 72.0) |
| AST, median (range), IU/L | 29.0 (6.00, 256) |
| Creatinine clearance, median (range), mL/min | 81.8 (27.9, 369) |
| Hemoglobin, median (range), g/L | 121 (75.0, 191) |
| Hematocrit, median (range) | 0.400 (0.20, 0.60) |
| Erythrocyte count, median (range), 10^12^/L | 4.1 (2.6, 6.9) |

*AST*, aspartate aminotransferase; *ECOG PS*, Eastern Cooperative Oncology Group performance status; *HCC*, hepatocellular carcinoma; *NSCLC*, non-small cell lung cancer.

**Supplementary Table S3** Parameter estimates of time-to-first edema event model

|  | Final TTE model | | Bootstrap predicted parameter  (5% and 95% percentile) | | |
| --- | --- | --- | --- | --- | --- |
|  | Value | RSE (%) | Mean | 5% | 95% |
| Base hazard | 0.00761 | 6.59 | 0.008 | 0.007 | 0.008 |
| Age covariate on base hazard | 0.0309 | 19.2 | 0.031 | 0.020 | 0.044 |

*RSE,* relative standard error; *TTE*, time-to-event.

**Supplementary Table S4** Albumin model parameter estimates

|  | Final model | | Bootstrap predicted parameter  (5% and 95% percentile) | | |
| --- | --- | --- | --- | --- | --- |
|  | Value | RSE (%) | Mean | 5% | 95% |
| Albumin baseline (g/L) | 37.9 | 0.627 | 37.857 | 37.491 | 38.370 |
| K_out_ days^-1^ | 0.0240 | 3.74 | 0.024 | 0.021 | 0.027 |
| AUC_50_ (µg·h/mL) | 0.215 | 24.5 | 0.167 | 0.024 | 0.327 |
| I_max_ | 0.261 | 2.21 | 0.260 | 0.244 | 0.276 |
|  |  |  |  |  |  |
| IIV albumin baseline | 0.132 | 5.07 |  |  |  |
| IIV K_out_ | 0.712 | 10.8 |  |  |  |
| IIV I_max_ | 0.615 | 7.97 |  |  |  |

*AUC_50_*_,_ half the maximum effect; *I_max_*, maximum inhibition; *K_out_*, first-order degradation rate constant of albumin; *RSE,* relative standard error.

**Supplementary Table S5** Model predicted steady-state ΔQTcF

|  | Mean C_max_ (ng/mL) | |  | 90% CI of ΔQTcF (msec)  Bootstrapped | |
| --- | --- | --- | --- | --- | --- |
| Tepotinib dose | Tepotinib | MCS2571109A | Predicted mean ΔQTcF | Lower bound | Upper bound |
| Predicted ΔQTcF at mean observed mean C_max_ of tepotinib at steady state | | | | | |
| 500 mg | 1,000.2^a^ | — | 2.00 | 0.48 | 3.75 |
| 1,400 mg | 1,818.5^a^ | — | 4.63 | 1.52 | 7.54 |
| Predicted ΔQTcF at mean C_max_ of tepotinib and MCS2571109A (with interaction term) at steady state | | | | | |
| 500 mg | 1,000.2^a^ | 319.3^a^ | 3.08 | 1.25 | 4.34 |
| 1,000 mg | 1,199.4^a^ | 384.4^a^ | 5.18 | 2.59 | 6.75 |
| Predicted ΔQTcF at mean observed mean C_max_ of tepotinib at steady state in the VISION cohort | | | | | |
| 500 mg | 1,236^b^ | — | 4.41 | 0.97 | 7.87 |
| Predicted ΔQTcF at mean C_max_ of tepotinib and MCS2571109A (with interaction term) at steady state in the VISION cohort | | | | | |
| 500 mg | 1,236^b^ | 416^b^ | 5.13 | 1.39 | 9.29 |

*QTcF*, QT interval corrected using Fredericia’s formula.

^a^Geometric Mean C_max_.

^b^Population PK mean C_max_.

**Supplementary Table S6** Maximum mean ∆QTcF by dose group and study number

| Study | Tepotinib dose | Regimen^a^ | Formulation | Maximum mean ∆QTcF (90% CI) | Post-dose time point |
| --- | --- | --- | --- | --- | --- |
| 001 | 60 mg | 1 | Capsule | 2.7 msec (-13.5 msec, 18.9 msec) | Cycle 1 Day 8, predose |
|  | 60 mg | 2 | Capsule | 14.7 msec (1.2 msec, 28.2 msec) | Cycle 1 Day 1, 4 hour |
|  | 100 mg | 2 | Capsule | 2.8 msec (-9.9 msec, 15.6 msec) | Cycle 1 Day 1, 24 hour |
|  | 115 mg | 1 | Capsule | -0.1 msec (-4.0 msec, 3.8 msec) | Cycle 1 Day 14, predose |
|  | 115 mg | 2 | Capsule | 5.7 msec (-0.4 msec, 11.8 msec) | Cycle 1 Day 1, 4 hour |
|  | 130 mg | 2 | Capsule | 16.8 msec (5.7 msec, 27.9 msec) | Cycle 1 Day 19, 4 hour |
|  | 500 mg | 3 | Capsule | 0.8 msec (-5.0 msec, 6.6 msec) | Cycle 1 Day 14, 10 hour |
|  | 500 mg | 3 | Tablet | 13.6 msec (-1.2 msec, 28.3 msec) | Cycle 2 Day 1, predose |
|  | 1,000 mg | 3 | Capsule | 6.4 msec (-3.3 msec, 16.2 msec) | Cycle 1 Day 8, predose |
|  | 1,400 mg | 3 | Capsule | 5.7 msec (-1.6 msec, 13.0 msec) | Cycle 1 Day 8, predose |
| 003 | 500 mg | - | Capsule | 7.3 msec (-2.2 msec, 16.8 msec) | Cycle 1 Day 14, 10 hour |
| 004 | 300 mg | - | Tablet | 1.5 msec (-11.2 msec, 14.2 msec) | Cycle 1 Day 15, predose |
|  | 500 mg | - | Tablet | 5.1 msec (0.3 msec, 9.8 msec) | Cycle 1 Day 15, predose |
|  | 1,000 mg | - | Tablet | -6.7 msec (-21.9 msec, 8.5 msec) | Cycle 1 Day 15, 4 hour |
| 005 | 500 mg | - | Tablet | -2.4 msec (-4.6 msec, -0.2 msec) | Cycle 1 Day 1, 4 hour |

*QTcF*, QT interval corrected using Fredericia’s formula.

^a^Study 001 consisted of three dosing regimens; regimen 1 consisted of 30 to 400 mg of micronized tepotinib capsules, once daily for 14 days followed by 7 days with no treatment; regimen 2 was 60 to 315 mg micronized capsules, once daily three times a week (Days 1, 3, 5) for 3 weeks, and regimen 3 consisted of continuous once daily treatment in cycles of 21 days at dose levels ranging from 300 to 1,400 mg.

**Supplementary Table S7** Categorical outlier analysis for QTcF parameter

| Study | Tepotinib dose | Regimen^a^ | Formulation | N | Parameter outlier criteria | | | | |
| --- | --- | --- | --- | --- | --- | --- | --- | --- | --- |
|  |  |  |  |  | >450 to ⩽480 msec value,  n (%) | >480 to ⩽500 msec value,  n (%) | >500 msec value,  n (%) | >30 to ⩽60 msec CFB,  n (%) | >60 msec CFB,  n (%) |
| 001 | 30 mg | 1 | Capsule | 3 | (0%) | (0%) | (0%) | (0%) | (0%) |
|  |  | 2 | Capsule | 2 | (0%) | (0%) | (0%) | (0%) | (0%) |
|  | 60 mg | 1 | Capsule | 6 | (0%) | (0%) | (0%) | 1 (16.7%) | (0%) |
|  |  | 2 | Capsule | 9 | 4 (44.4%) | 1 (11.1%) | (0%) | 2 (22.2%) | 1 (11.1%) |
|  | 100 mg | 1 | Capsule | 3 | 1 (33.3%) | (0%) | (0%) | (0%) | (0%) |
|  |  | 2 | Capsule | 6 | (0%) | (0%) | (0%) | (0%) | (0%) |
|  | 115 mg | 1 | Capsule | 12 | 2 (16.7%) | (0%) | (0%) | 1 (8.3%) | (0%) |
|  |  | 2 | Capsule | 13 | 2 (15.4%) | (0%) | (0%) | 1 (7.7%) | (0%) |
|  | 130 mg | 2 | Capsule | 6 | 1 (16.7%) | (0%) | (0%) | 2 (33.3%) | (0%) |
|  | 145 mg | 1 | Capsule | 3 | (0%) | (0%) | (0%) | 1 (33.3%) | (0%) |
|  | 175 mg | 2 | Capsule | 3 | (0%) | (0%) | (0%) | (0%) | (0%) |
|  | 215 mg | 1 | Capsule | 3 | 1 (33.3%) | 1 (33.3%) | (0%) | (0%) | (0%) |
|  | 230 mg | 1 | Capsule | 3 | (0%) | (0%) | (0%) | (0%) | (0%) |
|  | 300 mg | 1 | Capsule | 3 | (0%) | (0%) | (0%) | (0%) | (0%) |
|  | 300 mg | 3 | Capsule | 3 | 1 (33.3%) | (0%) | (0%) | (0%) | (0%) |
|  | 315 mg | 2 | Capsule | 4 | (0%) | (0%) | (0%) | (0%) | (0%) |
|  | 400 mg | 1 | Capsule | 3 | (0%) | 1 (33.3%) | (0%) | (0%) | (0%) |
|  | 500 mg | 3 | Capsule | 18 | 4 (22.2%) | 1 (5.6%) | (0%) | (0%) | (0%) |
|  |  |  | Tablet | 24 | 4 (16.7%) | 1 (4.2%) | 0 | 4 (16.7%) | 0 |
|  | 700 mg | 3 | Capsule | 3 | (0%) | (0%) | (0%) | (0%) | (0%) |
|  | 1,000 mg | 3 | Capsule | 7 | (0%) | (0%) | (0%) | 1 (14.3%) | (0%) |
|  | 1,400 mg | 3 | Capsule | 7 | 1 (14.3%) | (0%) | (0%) | (0%) | (0%) |
| 003 | 215 mg | - | Capsule | 3 | (0%) | (0%) | (0%) | (0%) | (0%) |
|  | 300 mg | - | Capsule | 3 | (0%) | (0%) | (0%) | (0%) | (0%) |
|  | 500 mg | - | Capsule | 6 | 2 (33.3%) | (0%) | (0%) | (0%) | (0%) |
| 004 | 300 mg | - | Tablet | 7 | 2 (28.6%) | (0%) | 1 (14.3%) | 1 (14.3%) | (0%) |
|  | 500 mg | - | Tablet | 57 | 13 (22.8%) | 1 (1.8%) | 1 (1.8%) | 6 (10.5%) | 1 (1.8%) |
|  | 1,000 mg | - | Tablet | 6 | (0%) | (0%) | (0%) | (0%) | (0%) |
| 005 | 300 mg | - | Tablet, film coated | 4 | (0%) | (0%) | (0%) | (0%) | (0%) |
|  | 500 mg | - | Tablet, film coated | 55 | 9 (16.4%) | 3 (5.5%) | 4 (7.3%) | 3 (5.5%) | 1 (1.8%) |

*CFB*, change from baseline; *QTcF*, QT interval corrected using Fredericia’s formula.

^a^Study 001 consisted of three dosing regimens; regimen 1 consisted of 30 to 400 mg of micronized tepotinib capsules, once daily for 14 days followed by 7 days with no treatment; regimen 2 was 60 to 315 mg micronized capsules, once daily three times a week (Days 1, 3, 5) for 3 weeks, and regimen 3 consisted of continuous, once daily treatment in cycles of 21 days at dose levels ranging from 300 to 1,400 mg.

**Supplementary Table S8** Mean observed QTcF and ΔQTcF values from the VISION study

| Visit  (Cycle/Day) | Timepoint | Observed QTcF | | | Change from Baseline (ΔQTcF) | | | |
| --- | --- | --- | --- | --- | --- | --- | --- | --- |
|  |  | N | Mean QTcF (msec) | SD  (msec) | N | Mean QTcF (msec) | SD  (msec) | 2-sided 90% CI (msec) |
| Cycle 1 Day 1 | Predose  (baseline) | 118 | 411 | 25.45 | Not applicable | | | |
|  | 4h Post-dose | 118 | 408.5 | 25.06 | 117 | -2.5 | 12.90 | (-4.43, -0.48) |
| Cycle 2 Day 1 | Predose | 102 | 410.2 | 21.48 | 100 | 1.9 | 17.15 | (-0.96, 4.74) |
|  | 4h Post-dose | 101 | 410.4 | 21.58 | 99 | 1.7 | 16.14 | (-1.00, 4.39) |

*CI*, confidence interval; *QTcF*, QT interval corrected using Fredericia’s formula; *SD*, standard deviation.

**Supplementary Table S9** Categorical analysis of QTcF values from the VISION study (*n=*120)

| Category, n (%) | At any  timepoint | At baseline | At post-baseline  timepoint |
| --- | --- | --- | --- |
| QTcF >450 msec to ≤ 480 msec | 9 (7.5%) | 3 (2.5%) | 8 (6.7%) |
| QTcF >480 msec to ≤ 500 msec | 0 | 0 | 0 |
| QTcF >500 msec | 1 (0.8%) | 1 (0.8%) | 1 (0.8%) |
| QTcF increase from baseline >30 msec to ≤60 msec | 9 (7.5%) | Not applicable | 9 (7.5%) |
| QTcF increase from baseline >60 msec | 0 | Not applicable | 0 |

*QTcF*, QT interval corrected using Fredericia’s formula.

**Supplementary Fig. S1** Time-to-event model for edema. Visual predictive check of final time-to-event model. The solid and dashed blue lines represent the Kaplan–Meier point estimate and 95% confidence interval, based on observed data; the shaded green areas represent the 95% confidence interval of the Kaplan–Meier point estimate, based on 200 replicate simulations.

**Supplementary Fig. S2** Observed individual maximum change from baseline serum creatinine concentration versus MCS2571109A AUC_24h_. Dots represent observations. The solid black line is a LOESS smooth. *AUC_24h_*, 24-hour area under the curve; *LOESS,* locally estimated scatterplot smoothing.


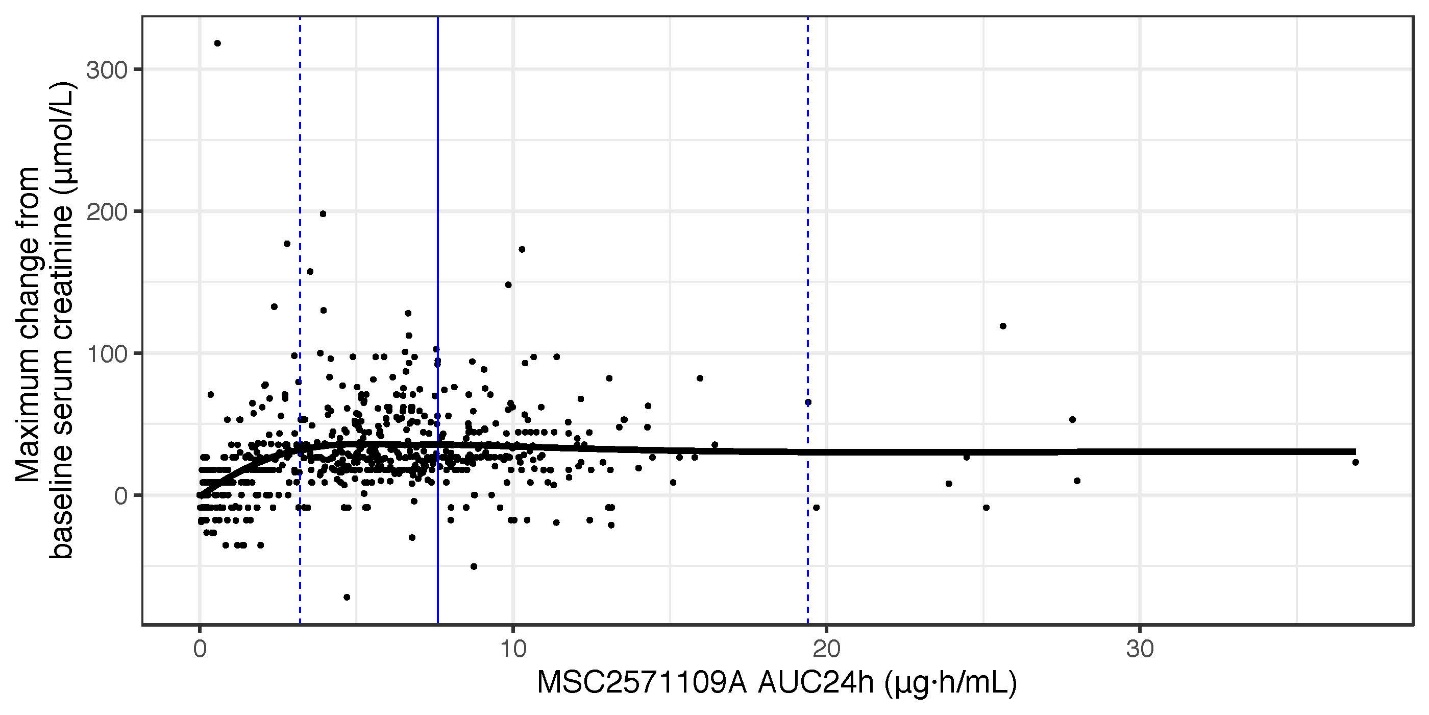


**Supplementary Fig. S3** Relationship between tepotinib exposure and treatment discontinuation due to an adverse event (data cut-off: July 1, 2020). Panels **a** and **b** present Kaplan–Meier analysis of temporary treatment discontinuation stratified according to (**a**) tepotinib and (**b**) MCS2571109A exposure quartile. Panels **c** and **d** present Kaplan–Meier analysis of time to permanent treatment discontinuation stratified according to (**c**) tepotinib and (**d**) MCS2571109A exposure quartile. In all panels, shaded areas represent 95% confidence intervals. *AE*, adverse event; *AUC*, area under the curve; *AUC_ss_*, area under the curve at steady state.

a

b

c

d

**Supplementary Fig. S4** Relationship between MCS2571109A exposure and grade ≥3 adverse event and dose reduction due to an adverse event (data cut-off: July 1, 2020). Panel **a** presents Kaplan–Meier analysis of time-to-first grade ≥3 AE stratified according to MCS2571109A exposure quartile. Panel **b** presents Kaplan–Meier analysis of time-to-first dose reduction due to an AE according to MCS2571109A exposure quartile. Shaded areas represent 95% confidence intervals. *AE*, adverse event; *AUC*, area under the curve; *AUC_ss_*, area under the curve at steady state.

a

b
